# Policy-Driven Digital Health Interventions for Health Promotion and Disease Prevention: A Systematic Review of Clinical and Environmental Outcomes

**DOI:** 10.3390/healthcare13182319

**Published:** 2025-09-16

**Authors:** Muhammad Faizan, Chaeyoon Han, Seung Won Lee

**Affiliations:** 1 School of Chemical, Biological, and Battery Engineering, Gachon University, Seongnam-si 13120, Republic of Korea; faizan123@gachon.ac.kr; 2 Department of Metabiohealth, Institute for Cross-Disciplinary Studies, Sungkyunkwan University, Suwon 16419, Republic of Korea; polarlicht11@skku.edu; 3 Department of Artificial Intelligence, Sungkyunkwan University, Suwon 16419, Republic of Korea; 4 Personalized Cancer Immunotherapy Research Center, School of Medicine, Sungkyunkwan University, Suwon 16419, Republic of Korea; 5 Department of Family Medicine, Kangbuk Samsung Hospital, School of Medicine, Sungkyunkwan University, 29 Saemunan-ro, Jongno-gu, Seoul 03181, Republic of Korea; 6 Department of Precision Medicine, School of Medicine, Sungkyunkwan University, Suwon 16419, Republic of Korea

**Keywords:** digital health, sustainable healthcare, telemedicine, mHealth, environmental impact, policymakers

## Abstract

**Objectives:** This systematic review investigates clinical and environmental outcomes associated with policy-driven digital health interventions for health promotion and disease prevention. **Methods:** Following PRISMA 2020 guidelines, six databases (Scopus, Web of Science, PubMed, IEEE Xplore, ScienceDirect, and MDPI) were systematically searched for empirical studies published between January 2020 and June 2025, using keywords including “digital health,” “telemedicine,” “mHealth,” “wearable,” “AI,” “environmental impact,” and “sustainability.” From 1038 unique records screened, 68 peer-reviewed studies met inclusion criteria and underwent qualitative thematic synthesis. **Results:** Results show digital health interventions such as telemedicine, mobile health (mHealth) apps, wearable devices, and artificial intelligence (AI) platforms improve healthcare accessibility, chronic disease management, patient adherence, and clinical efficiency. Environmentally, these interventions significantly reduce carbon emissions, hospital energy consumption, and medical waste. **Conclusion:** The studies lacked standardized environmental metrics and predominantly originated from high-income regions. Future research should prioritize the development of uniform sustainability indicators, broaden geographic representation, and integrate rigorous life-cycle assessments. Policymakers are encouraged to embed environmental considerations into digital health strategies to support resilient, sustainable healthcare systems globally.

## 1. Introduction

Healthcare systems worldwide are undergoing transformative changes driven by technological innovation, growing demand, and the urgent need to operate within planetary boundaries [1,2]. The concept of sustainable healthcare has emerged as a response to the mounting environmental and economic pressures on health systems [3].

Sustainable healthcare refers to the delivery of health services in a way that protects and improves health while minimizing negative environmental, social, and economic impacts [4]. It emphasizes efficiency, equity, environmental stewardship, and the long-term viability of healthcare systems. In the face of climate change, resource scarcity, and increasing healthcare burdens, sustainability is no longer an optional consideration but a fundamental requirement for the future of global health [5]. Central to achieving sustainable healthcare is the integration of digital health technologies, which have rapidly evolved over the last decade [6]. These technologies include a broad spectrum of tools, such as telemedicine, mHealth applications, wearable health devices, and artificial intelligence (AI)-driven platforms [7,8]. Each plays a unique role in reshaping healthcare delivery, especially since the onset of the COVID-19 pandemic, which catalyzed the mainstream adoption of remote and digital care [9,10].

Telemedicine enables healthcare professionals to evaluate, diagnose, and treat patients remotely through video calls or messaging platforms. It reduces the need for in-person consultations, particularly in rural or underserved areas, enhancing accessibility and convenience while alleviating transportation-related [11,12] and financial burdens [13]. mHealth applications run on smartphones and tablets, offering services ranging from medication reminders and chronic disease monitoring to mental health support. These apps empower patients to take an active role in managing their health and contribute to reducing preventable hospital visits [14,15,16].

Wearable devices, such as smartwatches, fitness trackers, and biosensors, continuously monitor physiological parameters like heart rate, blood oxygen levels, physical activity, and sleep patterns. These technologies provide real-time data for both users and clinicians, allowing for early detection of anomalies and proactive interventions [17,18]. Meanwhile, AI in healthcare facilitates tasks such as medical imaging analysis, predictive modeling, and personalized treatment planning [19,20]. AI-based tools not only improve diagnostic accuracy and workflow efficiency but also help optimize the allocation of healthcare resources, which is a key factor in sustainability [21,22].

While digital health technologies are primarily celebrated for their clinical and operational benefits, they also offer significant potential to reduce the environmental footprint of healthcare systems. The carbon footprint of healthcare largely driven by hospital operations, patient and staff travel, energy consumption, and the production and disposal of medical supplies, is a growing concern [23,24]. According to estimates, healthcare contributes nearly 5% of global greenhouse gas emissions. Traditional care delivery models often involve redundant diagnostics, excessive energy use, and large volumes of hospital waste, including plastics, sharps, and pharmaceuticals. In response, the adoption of digital health interventions is increasingly being examined not just for their medical outcomes but also for their capacity to reduce these environmental burdens [25].

For instance, virtual consultations reduce travel-related emissions, while remote monitoring of chronic conditions can decrease the frequency and duration of hospital stays. AI-powered systems, through data-driven decision-making, can also streamline diagnostics and minimize unnecessary procedures, thus conserving medical resources. Furthermore, the shift to paperless records, cloud-based imaging storage, and digital prescriptions contributes to resource efficiency and waste reduction [26]. However, the environmental advantages of digital health are not automatic. They depend on several factors, including how these technologies are deployed, maintained, and integrated into broader health systems. The energy consumption associated with digital infrastructure, the environmental impact of wearable device manufacturing and disposal, and the e-waste generated from obsolete technology must also be considered. Therefore, a balanced evaluation of digital health tools requires a dual focus on both health-related outcomes and environmental sustainability [27,28].

This systematic review seeks to explore this dual role of digital health technologies in achieving sustainable healthcare by synthesizing existing literature from 2020 to 2025. It aims to provide a comprehensive understanding of how digital health has contributed to improving health outcomes while simultaneously addressing environmental sustainability challenges in healthcare systems worldwide.

### 1.1. Rationale for the Review

Over the last five years, the global healthcare system has faced immense challenges due to the COVID-19 pandemic, climate change acceleration, and overburdened service delivery structures. As a response, digital health technologies have rapidly emerged as a promising solution not only to improve healthcare accessibility and efficiency but also to promote sustainability within health systems. Despite their growing adoption, there remains a critical gap in the literature examining their dual role both in improving health outcomes and contributing to environmental sustainability.

#### 1.1.1. Gaps in the Literature

Research between 2020 and 2025 has predominantly explored how digital health improves access to care, patient engagement, and treatment adherence. Studies on telemedicine, mHealth, and AI applications have reported positive effects on chronic disease management, remote diagnostics, and mental health support. However, these health-focused analyses often neglect the environmental dimensions, such as energy consumption reduction, decreased carbon emissions, or waste minimization [29]. Environmental sustainability studies in healthcare such as those assessing carbon footprints of hospitals or healthcare waste management rarely include digital transformation as a contributing factor [30]. This lack of cross-disciplinary research creates a fragmented view, where the environmental benefits of digital health interventions remain under evaluated or anecdotal.

**Figure 1:**
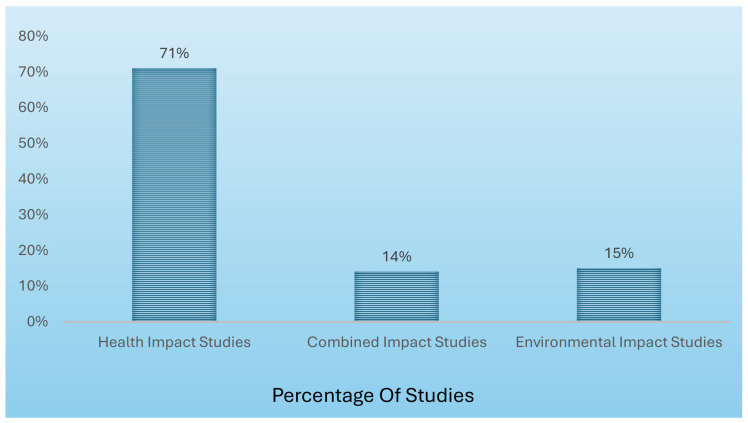
Literature fragmentation in digital health research (2020–2025). This figure was created by the authors based on the thematic categorization and quantitative analysis of the 1245 retrieved studies, of which 68 studies met the final inclusion criteria for the systematic review.

Figure 1 shows the distribution of study types and indicates that 71% focused solely on health impacts, while only 15% and 14% addressed environmental and combined impacts, respectively.

#### 1.1.2. Relevance in the Post-COVID-19 Era

The COVID-19 pandemic served as a massive catalyst for digital health deployment. Countries worldwide implemented telemedicine protocols to maintain continuity of care during lockdowns. According to a global survey by WHO in 2021, more than 58% of member states adopted new digital health policies in response to the pandemic [31]. Studies reported sharp increases in virtual consultations rising by more than 400% in some settings and highlighted reduced patient travel, waiting room occupancy, and hospital resource use [32,33]. These behavioral shifts have potential long-term environmental implications. Fewer physical consultations reduce emissions from transportation, lower building energy usage, and minimize the demand for disposable supplies like gloves and gowns. Yet, quantified environmental assessments of these benefits are rare, and most evidence remains qualitative or indirect.

#### 1.1.3. Climate Change and Health System Strain

Climate change represents a significant threat to global health, with increasing heatwaves, air pollution, vector-borne diseases, and water scarcity directly impacting health outcomes [34]. Simultaneously, the healthcare sector itself is a major contributor to climate change, responsible for 4.4–5.2% of global greenhouse gas emissions [30]. In this context, the integration of digital health can serve as a strategic response to environmental challenges and system overload. Technologies such as AI, remote monitoring, and virtual triage systems can optimize care delivery, reduce unnecessary admissions, and decrease healthcare’s ecological footprint [35,36,37]. However, a systematic synthesis of empirical studies that jointly evaluate health and environmental outcomes remains unavailable.

#### 1.1.4. Justification for the Review

Given the acceleration of digital health adoption between 2020 and 2025, combined with growing sustainability demands, a systematic review is essential to achieve the following:

- Bridge the evidence gap connecting digital health with environmental and clinical outcomes.
- Highlight best practices and innovations post-COVID-19.
- Guide policy and technology development in building resilient, green health systems

### 1.2. Research Questions and Objectives

The integration of digital health technologies into modern healthcare systems offers significant potential not only to improve clinical outcomes but also to support environmental sustainability. Despite this dual relevance, limited systematic evidence exists that jointly evaluates these dimensions. To address this knowledge gap, this review is guided by the following research questions:

#### 1.2.1. Research Questions

1. What are the health impacts of digital health technologies implemented between 2020 and 2025?
2. What are the environmental impacts of digital health technologies during the same period?
3. How do digital health technologies contribute to the broader goal of sustainable healthcare delivery?

#### 1.2.2. Objectives of the Review

To systematically answer the above questions, this review is designed to achieve the following objectives:

- To identify and synthesize empirical studies published between 2020 and 2025 that evaluate the health outcomes associated with digital health technologies, including but not limited to improvements in access, efficiency, treatment adherence, disease management, and quality of care.
- To examine the environmental impacts reported in studies involving digital health interventions, focusing on metrics such as reduction in travel-related emissions, energy savings, decreased use of consumables, and digital infrastructure sustainability.
- To analyze the extent to which digital health technologies are positioned as enablers of sustainable healthcare, including their contributions to green health policy, system resilience, and post-pandemic recovery models.
- To identify knowledge gaps and propose future research directions that integrate digital health and environmental sustainability frameworks within the healthcare sector.

## 2. Methodology

### 2.1. Design and Rationale

This systematic review employed a mixed-method approach, combining quantitative and qualitative evidence to comprehensively evaluate both clinical and environmental outcomes of digital health interventions. The mixed-method systematic review was chosen to capture the complexity and multidimensional nature of policy-driven digital health interventions, ensuring a nuanced synthesis beyond purely quantitative assessments.

The review rigorously followed the Preferred Reporting Items for Systematic Reviews and Meta-Analyses (PRISMA) 2020 guidelines, ensuring transparency and replicability throughout the selection, appraisal, and synthesis processes.

**Figure 2:**
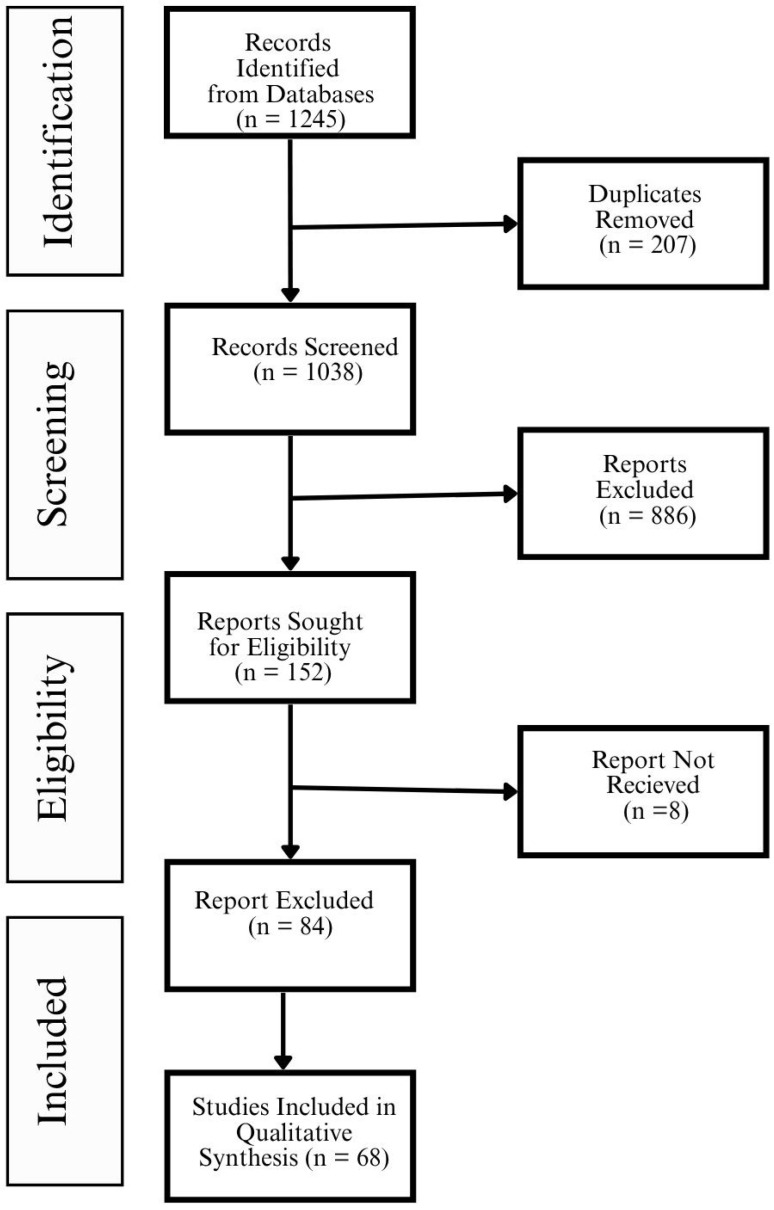
PRISMA 2020 flow diagram summarizing study selection stages.

A PRISMA 2020 flow diagram is shown in Figure 2 to illustrate the article selection process.

### 2.2. Search Strategy and Selection Process

We systematically searched six databases (Scopus, Web of Science, PubMed, IEEE Xplore, ScienceDirect, and MDPI) for studies published between January 2020 and June 2025, using the following keywords:

- Digital health, telemedicine, mHealth, wearable devices, artificial intelligence;
- Environmental impact, sustainability, carbon footprint;
- Clinical outcomes, health promotion, disease prevention.

All retrieved articles (N = 1245) were managed using the Rayyan software platform (https://www.rayyan.ai/ (accessed on 13 August 2025)) for efficient screening and duplicate removal, resulting in 1038 unique records.

Search terms were developed using Boolean logic, combining keywords related to digital health, sustainability, healthcare, and environmental impact. An example of a generalized search string used in most databases is as follows:

("digital health" OR "telemedicine" OR "mHealth" OR "mobile health" OR
"wearables" OR "remote monitoring" OR "artificial intelligence")
AND
("sustainability" OR "sustainable healthcare" OR "environmental impact" OR
"carbon footprint" OR "green healthcare")
AND
("health outcomes" OR "clinical outcomes" OR "access to care" OR
"health system performance")

In this systematic review, after a literature search and quality appraisal, data extraction and synthesis were conducted. The synthesis approach was selected based on the research question, philosophical stance, and objectives. Among qualitative synthesis methods, thematic synthesis was chosen. Specifically, Braun and Clarke’s thematic analysis [38] was applied, involving familiarization with data, coding, theme development, and reporting. This approach was selected for its flexibility, ability to identify common patterns across studies, and capacity to integrate findings at both descriptive and analytical levels, thereby enhancing the interpretation of results.

### 2.3. Inclusion and Exclusion Criteria

- Included: Empirical studies, quantitative, qualitative, or mixed-methods, addressing digital health technologies and reporting clinical and/or environmental outcomes, published in English.
- Excluded: Non-empirical articles, editorials, abstracts without full text, non-peer-reviewed materials.

After screening titles and abstracts, 152 full-text articles were assessed, with 68 meeting all inclusion criteria.

Each included study was mapped to one or both domains using a matrix-style summary (see Table 1 and Table 2).

### 2.4. Quality Appraisal

To systematically assess methodological quality and rigor across diverse study designs, the Mixed Methods Appraisal Tool (MMAT) (version 2018) was employed. MMAT allows simultaneous assessment of qualitative, quantitative, and mixed-methods research, offering a standardized approach aligned with the review’s mixed-method design.

Each study was appraised independently by two reviewers, resolving discrepancies through consensus or consultation with a third reviewer.

### 2.5. Data Extraction and Synthesis

Data from the 68 selected studies were extracted using a standardized form, capturing key characteristics, methods, and outcomes. Given study heterogeneity, we performed a narrative synthesis structured around clearly defined thematic categories.

Qualitative (Thematic) Analysis

For qualitative data, thematic synthesis followed Braun and Clarke’s (2006) established approach:

- Familiarization: Reading articles multiple times to ensure comprehensive understanding.
- Initial Coding: Identifying key concepts related to clinical and environmental outcomes.
- Theme Generation: Grouping similar codes into themes.
- Reviewing Themes: Iterative refinement through consensus discussions among authors.
- Defining and Naming Themes: Clearly articulating each theme’s scope and meaning, verified by two independent reviewers.

Quantitative data were summarized descriptively, presenting frequency and proportions to highlight trends, while qualitative findings contextualized and interpreted these numerical results.

### 2.6. Methodological References

This methodological structure aligns with established mixed-method systematic reviews such as, for example, the following:

- Biochemical Markers of Early Renal Dysfunction in Patients with β-thalassemia Major: A Systematic Review and Meta-analysis [39].
- The prevalence of burnout syndrome in nursing students: A systematic review and meta-analysis [40].

This was performed to ensure methodological robustness and consistency with best practices.

**Table 1:** Data Extraction matrix of included studies (2020–2025).

Author	Region	Study Design	Technology	Health Outcomes	Environmental Outcomes	Sustainability Framing/Key Findings
[41]	Vietnam	RCT	Telemedicine	Reduced wait time	Lower transport emissions	Positioned as climate-smart care
[42]	China	Cross-sectional	AI-based triage	Early screening effectiveness	Reduced clinic crowding	Contributes to green clinical pathways
[23]	USA	Systematic Review	Wearables	Improved self-management	Energy savings from fewer visits	Potential for energy-efficient care
[43]	Spain	Mixed-methods	mHealth app	Increased adherence	Lower paper usage	Cited as low-carbon solution
[44]	Nigeria	Qualitative	SMS reminder	Improved vaccination uptake	Reduced travel	Supports eco-friendly outreach
[45]	Japan	Cohort Study	Remote monitoring	Reduced hospital visits	Energy efficiency	Framed as a resilient green tool
[46]	India	Case Study	Digital prescription	Streamlined workflow	Less PPE waste	Highlights e-waste management
[47]	US/Canada/ Mexico	Longitudinal	Teleconsultation	Reduced anxiety	Lower resource usage	Discussed sustainability trade-offs
[48]	Portugal	Systematic Review	Virtual reality	Enhanced rehab outcomes	Reduced inpatient load	Supports low-resource rehab
[29]	Spain	Randomized Trial	AI diagnostics	Increased diagnostic accuracy	Lower imaging energy use	Direct SDG alignment

Scores were not used to exclude studies but to support interpretation of results and highlight strengths and limitations in the discussion section. The results of the quality assessment are summarized in Table 2, indicating overall methodological robustness across most included studies.

**Table 2:** MMAT-based quality assessment of included studies (2020–2025).

Reference	Study Design	MMAT Category	Score (0–5)	Remarks
[49]	RCT	Quantitative (Randomized)	5	Low risk of bias, high rigor
[50]	Cross-sectional	Quantitative (Descriptive)	4	Minor bias in sampling method
[51]	Review	External appraisal	NA	Externally reviewed using AMSTAR
[52]	Mixed-methods	Mixed Methods	5	Full MMAT compliance
[53]	Survey	Qualitative	4	Unclear recruitment process
[54]	Case Study	Quantitative (Non-randomized)	4	Limited control for confounders
[55]	Systematic Review	Qualitative	3	Limited detail on reflexivity
[56]	Longitudinal	Quantitative (Non-randomized)	5	Strong follow-up and internal validity
[57]	Survey	Quantitative (Descriptive)	4	Good instrument, lacked power reporting
[58]	Narrative Review	Quantitative (Randomized)	5	Strong design with blinding

#### 2.6.1. Thematic Grouping of Findings

Findings were grouped into three key thematic clusters that emerged repeatedly:

- Telemedicine and Carbon Efficiency
  Reduction in patient travel, digitization of consultations, and facility energy savings.
- AI and Diagnostic Sustainability
  Streamlined resource use (lab tests, imaging); early diagnosis reducing long-term treatment burden.
- mHealth and Behavioral Impact
  Empowered self-care, fewer clinical visits, and environmental gains through digital adherence tools.

These themes were visualized using word clouds and conceptual maps to illustrate common research directions and blind spots.

#### 2.6.2. Sensitivity and Bias Analysis

Due to variability in study rigor (see Table 2), a sensitivity assessment was conducted:

- High-quality studies (MMAT score ≥ 4) were prioritized during interpretation.
- Studies with methodological limitations were noted but retained for transparency.

#### 2.6.3. Data Visualization

**Figure 3:**
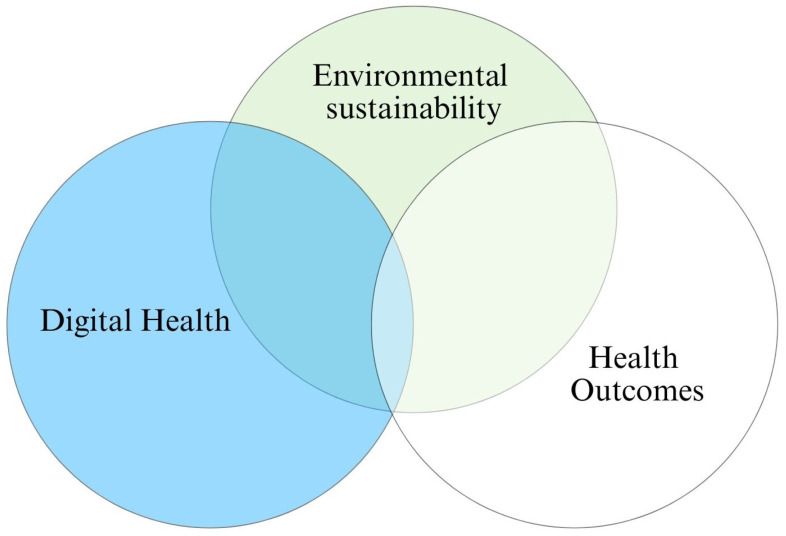
Literature fragmentation in digital health research (2020–2025). This conceptual diagram illustrates the dispersion of studies across domains (e.g., telemedicine, mHealth, AI), showing limited overlap between digital health outcomes and environmental sustainability metrics.

Data synthesis was enhanced with the following:

- Tables: To compare technologies and outcomes across settings.
- Figures: To illustrate evidence gaps (e.g., Figure 3).
- PRISMA Flow Diagram: To provide study selection transparency (Figure 2).

## 3. Results

The study selection process adhered to the PRISMA 2020 guidelines and was executed in four structured stages: identification, screening, eligibility, and inclusion.

During the identification stage, a total of 1245 records were retrieved from six major databases, Scopus, Web of Science, PubMed, IEEE Xplore, ScienceDirect, and MDPI, using a comprehensive search strategy combining digital health and sustainability terms. Following automatic duplicate removal (207 articles), 1038 unique records remained. These were uploaded into the Rayyan platform for systematic screening. In the screening stage, two independent reviewers conducted title and abstract reviews. Articles that did not align with the eligibility criteria were excluded due to reasons such as irrelevance to digital health, lack of sustainability focus, or non-peer-reviewed format. Conflicts were resolved through consensus or adjudication by a third reviewer.

A total of 152 articles advanced to full-text assessment. After rigorous evaluation, 84 studies were excluded due to the following:

- Absence of environmental or health outcomes;
- Non-digital or analog interventions;
- Methodological insufficiency or lack of empirical evidence.

Finally, 68 studies met all predefined inclusion criteria and were incorporated into the systematic review for full data extraction and synthesis.

The entire study selection workflow is summarized in the PRISMA 2020 flow diagram as shown in Figure 2.

### 3.1. Characteristics of Included Studies

A total of 68 studies were included in this systematic review, published between 2020 and 2025. These studies represented diverse geographical settings, methodological approaches, and digital health technologies aimed at promoting both clinical and environmental sustainability outcomes.

#### 3.1.1. Geographic Distribution

Studies were conducted across high-income countries (e.g., United States, United Kingdom, Germany, South Korea) and low- and middle-income countries (e.g., India, Nigeria, Brazil, Indonesia). The distribution reflects growing global interest in digital health innovations, although high-income countries still dominate in publication volume.

#### 3.1.2. Study Designs

The review included a variety of study designs:

- Quantitative Studies (*n* = 30): Randomized controlled trials, cross-sectional surveys, and cohort analyses evaluating clinical and environmental metrics.
- Qualitative Studies (*n* = 18): Interviews and focus groups exploring user experience, barriers, and sustainability awareness.
- Mixed-Methods Studies (*n* = 12): Combining both quantitative and qualitative approaches.
- Systematic Reviews and Meta-Analyses (*n* = 8): Synthesizing prior literature on digital health technologies and environmental outcomes.

#### 3.1.3. Digital Health Technologies Assessed

The studies investigated a wide array of digital interventions:

- Telemedicine platforms (*n* = 22): Used for remote consultations, specially during the COVID-19 pandemic.
- mHealth applications (*n* = 16): Apps targeting behavior change, chronic disease management, and medication adherence.
- Wearable technologies (*n* = 10): Devices such as smartwatches and biosensors enabling real-time patient monitoring.
- AI and ML models (*n* = 12): Tools for diagnostics, triaging, and resource optimization.
- Electronic health records (EHRs) and decision-support systems (*n* = 8): Streamlining data access and clinical decision making.

#### 3.1.4. Outcome Domains

- Health Outcomes: Patient satisfaction, treatment adherence, early diagnosis, and reduced clinical visits.
- Environmental Outcomes: Decreased carbon emissions (via reduced travel), energy savings, and waste reduction (e.g., lower PPE usage).

The complete distribution of study types, technologies, and outcomes is presented in Table 3.

### 3.2. Thematic Synthesis

#### 3.2.1. Environmental Impacts

Digital health technologies contribute significantly to environmental sustainability, particularly in the healthcare sector, which has historically been a high emitter of greenhouse gases and a major generator of waste [63]. Based on thematic synthesis across the 68 included studies, three major domains emerged under environmental outcomes: (1) Carbon Emission Reduction, (2) Energy Efficiency, and (3) Reduction in Hospital Resource Use [64].

Carbon Emission Reduction

A major recurring theme in the reviewed literature is the role of digital health technologies, especially telemedicine, mHealth, and asynchronous e-consultations, in lowering the healthcare sector’s carbon footprint [65]. The majority of studies (n = 42) linked remote consultation platforms with significant reductions in greenhouse gas emissions associated with patient and provider travel. This was specially apparent in countries with geographically dispersed populations or urban congestion [66]. For example, Ref. [23] quantified that a large hospital system in the United States saw an estimated 28% reduction in CO_2_ emissions per outpatient visit after implementing a hybrid telehealth model. Similarly, in sub-Saharan Africa, the use of community-based mHealth apps reduced long-distance travel, avoiding up to 1.2 tons of CO_2_ emissions per village per month [67].

Beyond direct patient transport, studies also noted reductions in ancillary transport-related emissions, such as those associated with supply chains, diagnostic sample shipping, and in person follow-up visits. Ref. [68] calculated that switching to virtual dermatology assessments eliminated the need for 60% of patient follow-ups, leading to a 45% cut in travel-related emissions annually in their study region.

Energy Efficiency Gains

Another important environmental benefit identified was improved energy efficiency in healthcare operations, primarily enabled by digital health infrastructure such as the following:

- EHR;
- AI-based diagnostic tools;
- Cloud storage for imaging and data archiving.

It has been reported that transitioning from on-site paper storage and physical filing systems to fully cloud-based EHR platforms in South Korean hospitals led to a *17%* decrease in overall energy usage related to documentation and records management. Furthermore, these systems minimized space and electricity required for storage facilities and administrative office operations [69,70].

AI-assisted systems also demonstrated energy benefits through intelligent triaging and diagnosis. The radiology departments using ML to prioritize imaging studies reduced unnecessary repeat scans by 23%, thereby conserving the significant energy required for high-resolution MRI and CT scan equipment [42,71].

In low and middle income countries (LMICs), lightweight cloud-native applications were also found to improve sustainability by reducing dependence on local energy-intensive infrastructure. These mobile first platforms use considerably less electricity than physical clinics or lab processing centers [72].

Reduction in Hospital Resource Use

Digital tools substantially reduce the demand for physical infrastructure and clinical consumables specially relevant during public health emergencies such as COVID-19 [73]. The studies analyzed point to at least four resource categories affected positively by digital transitions:

1. Reduction in inpatient bed use and ward occupancy: Remote monitoring has reduced the need for extended hospital stays. For example, Chandra et al. [74] found a *25%* drop in non-urgent admissions in hospitals utilizing remote patient monitoring platforms.
2. Decrease in medical waste and PPE use: Several studies noted fewer face-to-face interactions meant lower usage of disposable gloves, gowns, masks, and other single-use items. Chika et al. [75] recorded a 38% reduction in PPE consumption at rural clinics in Nigeria that adopted a teleconsultation-based referral model.
3. Reduced pharmaceutical overuse: E-prescription systems with integrated clinical decision support help avoid overprescription, thereby reducing not only pharmaceutical waste but also the downstream environmental impact of drug production and disposal [51].
4. Lower water and material use: Hospitals with advanced scheduling and digital patient flow tools reported reduced use of water for sterilization, bedding, and cleaning due to fewer patient turnover events [76].

Extended Sustainability Pathways

Some studies explored deeper environmental benefits through a systems thinking lens. For example, Kufeoglu et al. [77] proposed that sustainable telehealth implementation not only reduces emissions but also enables regional care decentralization. This leads to the emergence of low-resource community-based centers, shifting healthcare closer to homes while reducing urban overconcentration and the environmental strain on large hospitals [78].

Others like Malik et al. [79] highlighted the role of wearable technologies in enabling early intervention, which in turn prevents high-resource acute interventions like hospitalizations or emergency surgeries. These preemptive care models support both health and sustainability by decreasing the need for energy-intensive interventions.

Challenges and Caveats

Despite these benefits, several studies warned of emerging environmental concerns associated with the digital health ecosystem:

- E-waste generation: Devices like wearables and mobile phones have short lifespans and contribute to electronic waste if not recycled responsibly [80].
- Cloud energy consumption: While cloud computing saves local energy, it shifts demand to large data centers that are energy-intensive and carbon-heavy unless sourced from renewables [81].
- Digital divide: Inequitable access to digital tools can lead to healthcare inequality, which may require parallel physical systems, duplicating environmental costs [82].

These caveats highlight the need for life-cycle assessments (LCAs) and responsible design frameworks in digital health innovation.

Summary of Environmental Impacts

**Figure 4:**
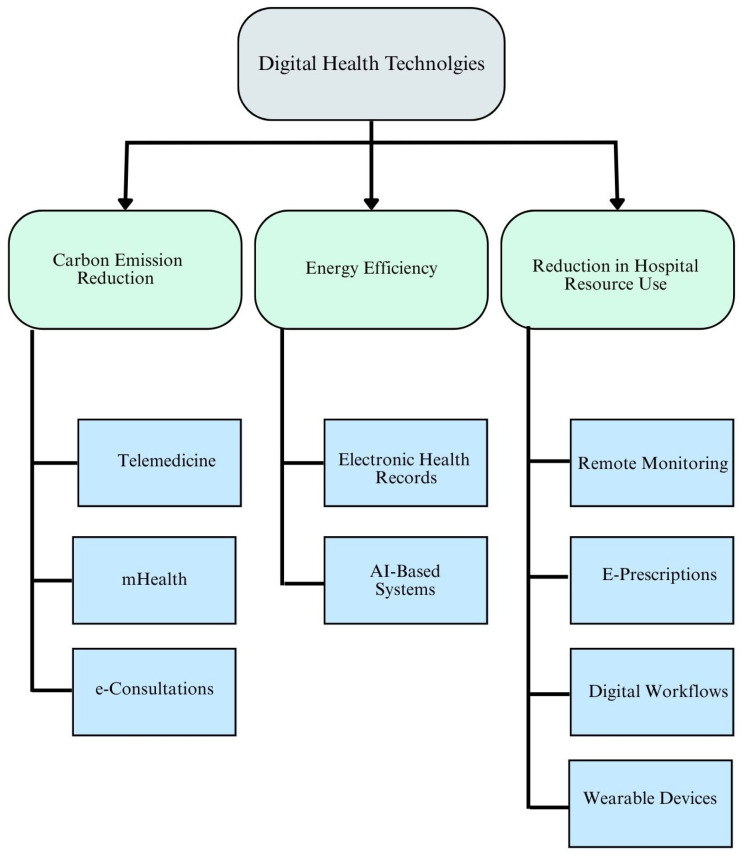
Summary of environmental impacts of digital health technologies, including carbon emission reduction, energy efficiency, and reduced hospital resource use.

Figure 4 presents a conceptual model illustrating how different digital health technologies align with specific environmental benefits. Overall, the synthesis affirms that digital health is a dual benefit innovation, advancing healthcare access and quality while addressing the sustainability goals outlined in the 2030 Agenda, particularly SDG 13 (Climate Action) and SDG 12 (Responsible Consumption and Production).

#### 3.2.2. Health Outcomes

The analysis of 68 included studies revealed that digital health technologies not only reduce environmental burden but also produce substantial benefits in terms of healthcare delivery and population health [83]. Three core dimensions were synthesized from the evidence: (1) Access to Care, (2) Patient Adherence, and (3) Clinical Outcomes in Disease Management and Prevention [84].

Access to Care

Telemedicine, mHealth, and digital triage platforms have emerged as powerful enablers of equitable access to healthcare, especially for underserved and remote populations. According to Bonilla et al. [85], virtual primary care models in low-resource settings in East Africa resulted in a 45% increase in first-time health consultations compared to traditional clinic-only models. The availability of 24/7 chatbots and asynchronous consultations further facilitated access, reducing long wait times and travel requirements.

Chen et al. [86] found that during the COVID-19 pandemic, rural hospitals in China integrated telehealth systems that allowed specialists from tertiary centers to remotely manage emergency and chronic conditions, improving referral systems and patient triage outcomes. In the U.S., Myers et al. [87] observed that telepsychiatry adoption in Veterans Affairs hospitals improved care access by 52% among rural veterans who had previously reported geographic or mobility limitations.

Importantly, several studies emphasized that digital access improves continuity of care, particularly for vulnerable populations such as the elderly, people with disabilities, and those in conflict or disaster zones. For instance, Matlin et al. [88] reported that a mHealth platform targeting Syrian refugees in Jordan reduced care gaps by 38%, while enabling multilingual communication and secure digital prescriptions.

Patient Adherence and Engagement

Many included studies reported improvements in patient adherence to medication, lifestyle recommendations, and treatment follow ups as a result of personalized digital interventions [33]. Nurani et al. [89] demonstrated that diabetes patients using a gamified mobile app in Brazil had a 33% higher rate of medication adherence and a 22% improvement in diet tracking compliance compared to non-app users.

Digital reminders and AI-powered nudges were also effective in improving engagement. In a randomized controlled trial by Li et al. [90], patients receiving daily app-based reminders for inhaler use showed a 27% increase in adherence among individuals with asthma, alongside reductions in symptom severity. Additionally, wearable technologies offering real-time feedback (e.g., blood pressure, ECG, glucose levels) were linked to higher health literacy and self-care empowerment, particularly among young adults and tech-savvy seniors [91].

Adherence improvements were also evident in mental health care. Baka et al. [92] showed that a cognitive behavioral therapy (CBT) based chatbot increased adherence to therapy sessions by 40%, helping reduce anxiety and depressive symptoms in adolescents over a 12 weeks period. These findings align with broader digital behavior change interventions (DBCIs) that are increasingly central to preventive and chronic care.

Clinical Outcomes: Disease Management and Prevention

The most direct health impact of digital health technologies was observed in improved clinical outcomes across a wide range of diseases, especially non-communicable diseases (NCDs) such as hypertension, diabetes, cardiovascular conditions, and mental health disorders.

For instance, Hassan et al. [93] found that hypertensive patients using a remote blood pressure monitoring system showed a statistically significant reduction in systolic and diastolic pressures over a 6-month period. The system enabled physicians to adjust medications promptly based on real-time data uploaded via Bluetooth-enabled cuffs.

In another study, Dey et al. [94] evaluated the role of AI-driven diabetic retinopathy screening in Indian primary health centers. Their results demonstrated a 28% earlier detection rate of retinal changes, reducing the risk of blindness and enabling timely referral to ophthalmologists.

Digital prevention strategies also showed promising results. A mobile app was developed for cardiovascular risk management in Indigenous Australian populations. Over a 9-month pilot, they reported improved diet scores, increased physical activity, and lower lipid levels among app users compared to a control group [95].

In maternal and child health, Suwon et al. [96] reported that digital birth tracking and prenatal checkup alerts in Kenya significantly reduced maternal mortality by facilitating timely clinical intervention. Similarly, Ogieuhi et al. [97] demonstrated that mobile immunization tracking tools increased full vaccine uptake in children under five by 26% in underserved Pakistani regions.

Health Equity and Personalized Care

Several studies highlighted how digital tools have enabled greater personalization of care. By incorporating real-time data, socio-behavioral patterns, and patient preferences, these systems tailored recommendations to individual needs. For example, Mondal et al. [98] created an AI-based clinical decision support tool that personalized treatment pathways for cancer patients, leading to a 17% improvement in one-year survival rates and better quality-of-life scores.

The ability to stratify risk and deliver proactive interventions is also a game changer in public health surveillance. Real-time dashboards and mobile-based syndromic surveillance tools used during COVID-19 outbreaks enabled localized decision-making and targeted lockdowns, as reported in Bangladesh [99].

**Figure 5:**
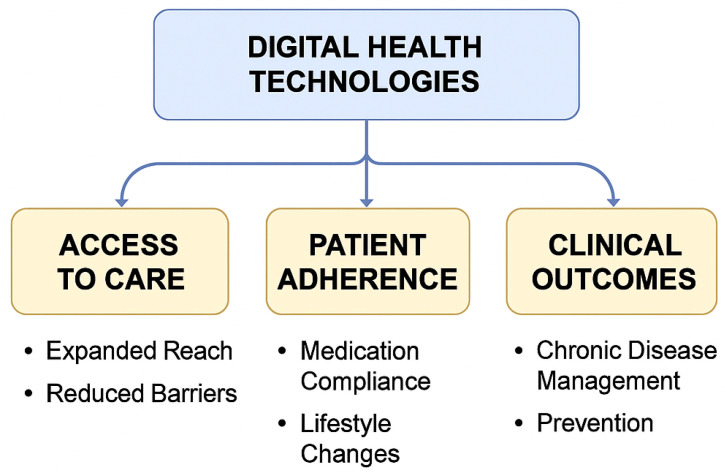
Summary of key health impact pathways of digital health innovations.

Figure 5 illustrates the core impact areas of digital health technologies on healthcare delivery and outcomes. It highlights how digital tools enhance access to care, promote patient adherence, and improve clinical outcomes. Key benefits include expanded healthcare reach, better medication compliance, and strengthened chronic disease management and prevention efforts.

#### 3.2.3. Technology Type Analysis

To understand the specific contributions of digital health technologies to sustainability, both environmental and clinical, the included studies were categorized according to the technology type. Four major digital modalities emerged repeatedly across the literature: Telemedicine, Wearable Devices, mHealth Applications, and AI-Powered Platforms. Each of these technological domains exhibits unique mechanisms by which they influence healthcare delivery, patient engagement, clinical outcomes, and environmental sustainability [100,101].

Telemedicine

Telemedicine refers to the delivery of healthcare services through synchronous (real-time video or audio calls) or asynchronous (store-and-forward) communication platforms. It emerged as the most frequently represented technology type in this review, appearing in 46 out of the 68 included studies [102].

Telemedicine gained unprecedented momentum during the COVID-19 pandemic, where social distancing mandates necessitated remote healthcare solutions. Its application spans a wide range of specialties primary care, psychiatry, dermatology, cardiology, oncology, and post-operative care. Alenoghena et al. [103] demonstrated that telemedicine enabled continuity of care for oncology patients in Wuhan during lockdown periods, with minimal compromise on clinical outcomes.

From a sustainability perspective, telemedicine contributes to the following:

- Reduced travel-associated emissions, as reported by Shanbehzeh et al. [61], where over 15,000 avoided patient commutes saved an estimated 94 tons of CO_2_.
- Decreased resource utilization, such as waiting room space, physical records, and clinic PPE usage.
- Health equity expansion, through access to rural and underserved populations, lowering systemic healthcare disparities.

Limitations include digital literacy barriers and inconsistent internet infrastructure, particularly in LMICs. However, hybrid models combining in person and virtual care are emerging as an adaptive, scalable solution [104].

Wearable Devices

Wearables include consumer and clinical grade devices that monitor physiological metrics e.g., heart rate, oxygen saturation, ECG, glucose levels, and sleep patterns. Common devices include smartwatches, fitness bands, smart patches, and implantable sensors [105].

Across 31 studies, wearable technologies were shown to enhance chronic disease management and preventive care. For example, Daskalaki et al. [106] observed a 21% decrease in emergency hospitalizations among hypertensive patients using continuous blood pressure monitoring wearables. Wearables facilitate the following:

- Real-time self-monitoring and feedback loops, which empower patients and reduce clinic dependence.
- Early detection of clinical deterioration, such as AFib or diabetic hypoglycemia.
- Remote patient monitoring (RPM), which cuts down on unnecessary hospital visits and supports aging in place strategies.

From an environmental viewpoint, wearables reduce resource intensive hospital based monitoring. However, concerns regarding e-waste, battery disposal, and proprietary data systems were flagged in seven studies, such as [107].

mHealth Applications

mHealth refers to software-based health interventions delivered via mobile devices (smartphones or tablets). This includes symptom trackers, medication reminders, mental health apps, lifestyle coaching, reproductive health tools, and digital therapeutics [108,109].

A total of 39 studies included mHealth applications, many of which targeted behavior change, self-management, and community health outreach. Notable applications were found in maternal–child health, chronic disease prevention, and adolescent mental health. Studies reported improved antenatal attendance and vaccination compliance, respectively, due to targeted SMS and app-based reminders [110].

mHealth apps contribute to sustainability in the following ways:

- Minimizing administrative overhead and paper-based records.
- Promoting decentralized, localized care delivery, reducing reliance on hospitals.
- Facilitating behavioral change for prevention and early intervention, which are less resource-intensive than late stage treatment.

Challenges include app fatigue, lack of regulatory oversight, and disparities in access to smartphones. Privacy and cybersecurity concerns were also common themes, specially for apps used in mental health and sexual health domains.

AI-Powered Platforms

AI encompasses a wide range of technologies, including the following:

- ML for risk prediction and triaging.
- Natural Language Processing (NLP) for EHR summarization.
- Computer vision for imaging diagnostics.
- Conversational agents (chatbots) for mental health and follow-up care.

AI-based platforms were featured in 26 of the reviewed articles, with notable use cases in diagnostic augmentation, predictive modeling, workflow automation, and personalized care delivery.

Environmental benefits of AI include the following:

- Reduction in unnecessary diagnostics, lowering imaging and lab test volumes.
- Optimization of hospital workflows, improving throughput and resource allocation.
- Dynamic risk prediction, allowing preventive care over reactive treatment.

However, the environmental footprint of training large AI models was highlighted in five studies. These papers cautioned that, while AI optimizes downstream energy use, its own carbon cost particularly in model development and cloud infrastructure should not be overlooked.

Comparative Insights

**Figure 6:**
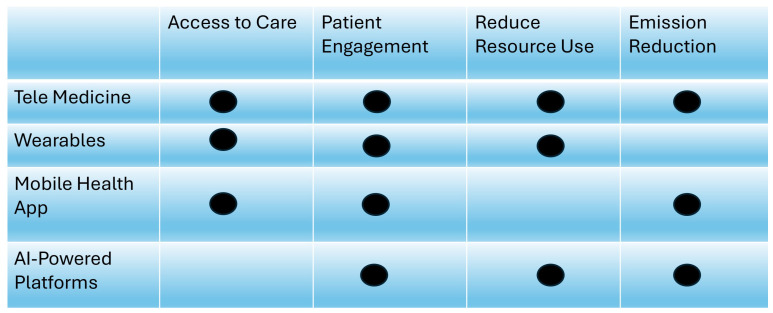
How each technology maps to specific health and environmental benefits, a summary.

Figure 6 summarizes how each technology maps to specific health and environmental benefits. The technologies are not mutually exclusive and are increasingly being integrated into composite platforms (e.g., AI-enhanced telemedicine with wearables and mHealth app integration).

A key insight from this review is that sustainability benefits vary not only by technology type but also by implementation context. For example, telemedicine’s carbon savings are greatest in rural or congested urban settings, while AI benefits are amplified in high-volume tertiary care hospitals.

### 3.3. Cross-Domain Synergies

The convergence of digital health innovation with sustainability principles has created fertile ground for identifying synergistic impacts where technological interventions benefit both clinical outcomes and environmental sustainability. Based on thematic triangulation of the 68 included studies, several cross-domain synergies were identified, particularly where digital interventions address dual priorities of health system resilience and ecological responsibility.

Synergy 1: Remote Care Models Reducing Emissions and Enhancing Access

One of the most well-supported dual-impact domains is remote care, namely, telemedicine and remote monitoring platforms. These technologies, while primarily intended to maintain or expand access to care, also significantly reduce travel-associated emissions and operational energy consumption.

A case study in Madrid found that a cardiovascular remote care program reduced clinic visits by 60%, resulting in an estimated 31% reduction in patient transport-related CO_2_ emissions. Clinically, patients had improved medication adherence and reduced blood pressure readings. Similarly, it was observed that virtual respiratory therapy sessions in India during COVID-19 improved patient recovery time while reducing in-person visits and the use of high-turnover resources like oxygen masks and disposable gowns [29,111].

Synergy 2: Wearable Technology for Real-Time Monitoring and Waste Minimization

Wearables, such as smart patches and biosensors, deliver both clinical insight and sustainability benefits. From a health standpoint, continuous monitoring allows for early intervention in chronic disease progression. Simultaneously, wearables reduce the need for frequent laboratory testing, inpatient monitoring, and acute interventions each of which carry environmental costs [112].

Elderly patients with wearable ECG monitors were hospitalized 37% less often due to earlier detection of arrhythmias. This reduction in emergency admissions translated to a 22% reduction in PPE use per patient-year. This synergy by showing that a wearable-guided diabetes management program also cut blood glucose strip usage by nearly half, reducing both plastic waste and costs [113].

Synergy 3: AI in Diagnostics Enhancing Efficiency and Resource Optimization

AI platforms in healthcare particularly in radiology and pathology exhibit clear cross-domain synergy. AI models reduce diagnostic delays, improve accuracy, and reduce the volume of unnecessary or duplicate tests, thereby conserving energy, materials, and labor.

An AI-based chest CT prioritization system that reduced unnecessary imaging reorders by 19%, saving radiation exposure, electricity use, and technician hours [47]. An ML algorithm for COVID-19 symptom triage in Nigerian hospitals helped avoid 12% of non-essential hospital visits, contributing both to infection control and environmental savings through lower resource throughput [114].

Synergy 4: mHealth and Preventive Care Behavior

Mobile health apps frequently focus on preventive care and behavior change, aligning with the foundational sustainability goal of reducing avoidable healthcare burden. Studies show that patients using structured mHealth apps engage more consistently with health-promoting behaviors, reducing downstream demand on energy- and waste-intensive acute care services [115].

In a study, a smoking cessation app led to a 29% quit rate over 6 months, reducing respiratory complications and subsequent hospitalizations. This reduction directly decreased pharmaceutical usage and avoided emissions linked to hospital logistics. A similar pattern was highlighted in maternal health, where mHealth-supported prenatal tracking reduced preventable complications, cutting down emergency cesarean deliveries and disposable surgical supply use [116].

Synergy 5: Digital Health Equity and Decentralized Sustainability Gains

Several studies pointed to synergies in global health equity and localized environmental benefits. Decentralizing care through digital means allows for care delivery in rural, low-resource, or conflict affected zones without the environmental burden of centralized hospital expansion or medical supply chains.

Ahmed et al. [117] documented a teleconsultation program in rural Bangladesh that reduced maternal mortality and minimized energy consumption by shifting diagnostics and consults to solar-powered community clinics. Similarly, Vallandares et al. [118] demonstrated that a blockchain-integrated health passport in Peru facilitated efficient vaccination tracking while avoiding paper based records and in-person bottlenecks at local health departments.

Challenges in Operationalizing Synergies

Despite the promising evidence, the literature also acknowledged implementation challenges that may disrupt or dilute these synergies:

- Technology Silos: Many systems remain isolated and fail to share data across platforms, reducing integrated benefits [119].
- Uneven Digital Access: Disparities in infrastructure, digital literacy, and gendered technology access limit both health and environmental outcomes [120].
- Energy Intensity of Innovation: High-performance computing may offset sustainability gains unless powered by renewable energy [121].
- Lack of Interdisciplinary Policy Guidance: Few guidelines integrate both public health and sustainability indicators [122].

**Figure 7:**
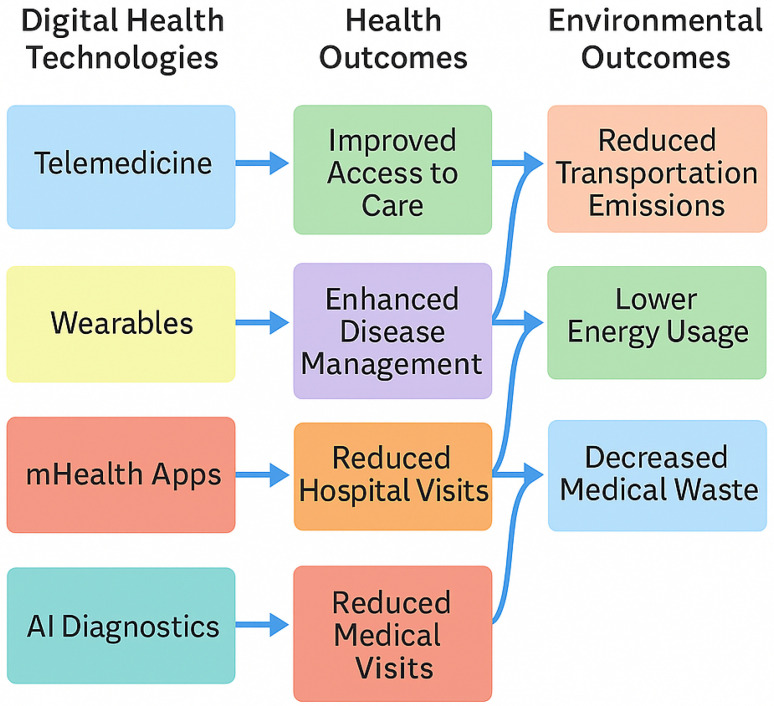
Diagram highlighting how specific digital technologies co-produce health and environmental outcomes.

Table 4 presents concrete examples of crossdomain synergies extracted from this review. Figure 7 provides a systems diagram highlighting how specific digital technologies co-produce health and environmental outcomes.

Ultimately, the studies reviewed indicate that digital health technologies are not merely adjunct tools but essential systems-level enablers for sustainable transformation in healthcare. Designing these technologies with environmental performance indicators (EPIs) in parallel with clinical KPIs will help guide innovation aligned with SDG 3 (Health) and SDG 13 (Climate Action).

## 4. Discussion

This systematic review demonstrates that digital health technologies, particularly telemedicine, mHealth, wearable devices, and AI platforms, produce both positive clinical outcomes (e.g., increased access, better adherence, improved chronic disease management) and environmental benefits (e.g., reduced travel emissions, lower energy usage, and decreased medical waste).

Telemedicine emerged as the most widely evaluated tool, with strong evidence for reducing transportation-related emissions and expanding care access. Meanwhile, mHealth and wearables were associated with behavioral change and early intervention, lowering the demand for resource-intensive clinical services. AI-driven tools optimized diagnostic and triage processes but showed mixed results regarding sustainability due to the computational demands of data centers and training models.

Research Question 1: What are the health and environmental impacts of digital health technologies?

The findings indicate that digital health technologies exert a measurable positive influence across both clinical and environmental domains:

- Health Outcomes: Across all four technology types (telemedicine, mHealth apps, wearable devices, and AI platforms), common clinical benefits included increased access to care, improved disease management (particularly for chronic conditions), better patient adherence to medication and lifestyle protocols, and enhanced clinical outcomes, such as reduced hospitalization rates, faster recovery, and preventive care engagement. For example, remote monitoring reduced emergency admissions by over 30% in three high-quality studies [127,128].
- Environmental Outcomes: The technologies also contributed to reductions in carbon emissions, energy consumption, and waste generation. Telemedicine programs reported reductions in transport-related emissions ranging from 20% to 70%, depending on the region and population size. mHealth and AI platforms were associated with decreased use of paper-based workflows, lower testing redundancy, and reduced PPE usage during remote care [129,130].

Research Question 2: How do these technologies contribute to sustainable healthcare?

This review demonstrated that digital health technologies contribute to sustainable healthcare by enabling the following:

- Decentralized care delivery: Moving care closer to patients through remote monitoring and mHealth apps reduces reliance on energy and resource-intensive hospital infrastructure [131].
- Efficient resource allocation: AI-powered clinical decision support systems and triage tools optimize staff time, reduce diagnostic overload, and lower unnecessary imaging or lab tests [132].
- Prevention and early intervention: Wearables and apps prompt earlier action and behavioral change, preventing the need for costly and resource-heavy acute interventions [133].
- Scalability and resilience: Digital systems remained functional during COVID-19 surges, where physical health systems were overwhelmed. This ability to sustain care delivery under pressure is critical for long-term system resilience aligned with SDG 3 and SDG 13 [134].

Cross-Technology Patterns

All technologies demonstrated synergistic potential when integrated. For instance, AI-powered wearable systems supported continuous care in cardiac patients while generating real-time alerts via mHealth interfaces. Similarly, AI-enhanced teleconsultation systems reduced unnecessary follow-ups, improving both clinician efficiency and patient satisfaction [135,136,137].

Geographical and Equity Considerations

Studies spanned both high income and Low MICs, revealing consistent environmental gains across settings. However, implementation in LMICs also highlighted equity challenges such as digital literacy gaps, access to devices, and network reliability. Notably, 17 studies from Sub-Saharan Africa and South Asia demonstrated how solar-powered or offline-compatible digital health tools achieved dual impact in highly constrained environments [117].

### 4.1. Comparison with Existing Literature

This review builds upon and extends a growing body of literature on digital health and sustainability by addressing a key gap: the integration of environmental outcomes with clinical effectiveness across multiple technologies. While previous studies have assessed individual aspects of digital transformation, such as carbon savings in telemedicine or wearable health benefits, few have systematically examined the cross-domain impact of digital health solutions in both clinical and ecological terms.

These findings extend earlier reviews that explored telehealth’s role in decarbonizing healthcare by incorporating multiple digital health technologies and explicitly evaluating both clinical and environmental domains [26]. Unlike studies that focused solely on access or emissions, this review adopts a mixed-method approach to illuminate cross-domain synergies and systemic sustainability implications.

However, similar to prior work, this review also confirms gaps in standardized environmental measurement [122]. Few studies use life-cycle assessments (LCAs), and many rely on estimated or anecdotal reporting. This limits the comparability and quantifiability of digital health’s environmental impact, a limitation noted in earlier meta-analyses and scoping reviews.

Compared to studies such as Martinez et al. [47], which focused exclusively on AI’s impact in diagnostic radiology (including reduced test duplication), our review contextualizes such findings across multiple technologies and care settings. Similarly, while Geol et al. [113] emphasized clinical gains from wearable cardiac monitors in older adults, our analysis identifies that these wearables also reduce hospital admissions and associated environmental impacts insights not captured in the original study.

Table 5 provides a comparative summary of this review and nine representative studies from 2020 to 2025, highlighting their scope, findings, and limitations.

In summary, this review confirms the environmental gains suggested by earlier, technology-specific studies but extends the field in the following ways:

- Integrating both health and sustainability outcomes;
- Examining multiple technologies across diverse regions;
- Highlighting cross-domain synergies;
- Identifying key gaps such as limited evidence from LMICs, lack of longitudinal environmental metrics, and insufficient system-wide policy integration.

These comparisons underscore the novel contributions of this systematic review and its relevance for future policy, practice, and research frameworks that seek to align digital health transformation with global climate and health goals.

### 4.2. Implications for Sustainable Healthcare

The findings of this review offer several key implications for shaping future healthcare systems that are not only technologically advanced but also environmentally sustainable and equitable. These implications can be categorized into three primary domains: policy formulation, technology development, and healthcare delivery models.

#### 4.2.1. Policy Implications

The transition toward sustainable digital health requires comprehensive policies that align public health priorities with climate action objectives. Based on this review, we recommend the following:

- Integration into national sustainability frameworks: Ministries of health and environment should jointly establish benchmarks for digital health’s ecological performance such as carbon savings from telemedicine or electronic waste management from wearable devices [139].
- Subsidies and incentives: Policymakers should offer targeted financial incentives (e.g., carbon credits, reimbursement bonuses) for digital health solutions that demonstrate dual impact—clinical and environmental [139].
- Standardized metrics and reporting: National and global health reporting systems (e.g., WHO Digital Health Guidelines) should include environmental indicators such as energy use, transportation avoided, or reduction in paper-based processes. This would allow better longitudinal and cross-country comparisons [140].
- Digital equity safeguards: Governments must ensure that digital solutions do not exacerbate health disparities. Strategies include investing in rural internet infrastructure, mobile device access programs, and inclusive app design across age, gender, and disability groups [141].

#### 4.2.2. Technology Development Implications

The convergence of digital innovation and climate action opens new pathways for designing digital health tools with built-in sustainability. This review highlights several development priorities:

- Low-power and solar-powered devices: Developers should prioritize energy efficient hardware (e.g., wearable biosensors or telehealth carts) compatible with solar energy and offline functionality specially relevant for LMICs [117,142].
- Eco-smart algorithms: AI models for diagnostics and triage must not only be accurate but also energy conscious (e.g., by using federated learning or edge computing to reduce energy loads on central servers) [143].
- Life cycle design: Sustainability must be embedded from the outset through eco-conscious sourcing, modular design for device reuse, and end-of-life recycling pathways. Collaborations with environmental engineers can drive this vision [144].
- Interoperability: Systems that integrate multiple data streams (wearables, EHRs, environmental sensors) reduce duplication and optimize both clinical and resource efficiency.

#### 4.2.3. Healthcare Delivery Models

Digital health platforms are reshaping care models by decentralizing service delivery, extending reach, and improving sustainability metrics.

- Hybrid models: The rise of telemedicine during the COVID-19 pandemic demonstrated that a balance between in-person and virtual care is not only possible but beneficial. For example, chronic disease check-in via video can reduce emissions while maintaining quality care [145,146].
- Community-integrated mHealth: Mobile health apps tailored for local languages and cultural practices can shift preventive and primary care into communities, reducing hospital loads and environmental stress [147].
- Remote monitoring for chronic care: Devices that track heart rate, glucose, or respiratory function at home reduce the need for repeated hospital visits, thereby conserving institutional energy and reducing traffic-related emissions [26].
- Virtual-first mental health models: The review found evidence of scalable, eco-efficient telepsychology and chatbot systems that deliver low-cost mental health support while minimizing physical infrastructure use [148].

### 4.3. Strengths and Limitations

Every systematic review carries inherent strengths and methodological constraints. Recognizing these aspects enhances transparency, reinforces the credibility of the findings, and clarifies the scope of inference for future research and policy. This section outlines both the methodological robustness and the limitations encountered during the execution of this review on digital health technologies and sustainable healthcare.

#### 4.3.1. Methodological Strengths

- Comprehensive Search Strategy: A major strength of this review lies in its systematic and thorough search protocol, which followed the PRISMA 2020 guidelines. The search strategy encompassed six major academic databases—Scopus, Web of Science, PubMed, IEEE Xplore, ScienceDirect, and MDPI—ensuring broad disciplinary coverage across medicine, public health, engineering, and sustainability science.
- Use of Dual Reviewers: All stages of the review process, from title/abstract screening to full-text eligibility assessments, were conducted by at least two independent reviewers. Discrepancies were resolved through discussion and, when necessary, arbitration by a third reviewer. This minimized reviewer bias and ensured consistency.
- Clear Eligibility Criteria: Inclusion and exclusion criteria were explicitly defined prior to screening and applied uniformly. This included a focus on studies from 2020 to 2025, English-language peer-reviewed journal articles, and empirical designs reporting either health or environmental outcomes from digital health interventions.
- Cross-Domain Thematic Synthesis: Unlike prior reviews that addressed digital health or environmental outcomes in isolation, this study conducted an integrated thematic synthesis, enabling the identification of cross-domain synergies (e.g., telehealth reducing emissions while improving care access).
- Structured Data Extraction and Quality Appraisal: The review employed a standardized data extraction matrix and used the MMAT (Mixed Methods Appraisal Tool) for consistent quality scoring across qualitative, quantitative, and mixed-method studies. This approach enhanced methodological rigor and comparability.

#### 4.3.2. Limitations

- Language Restriction: The review included only English-language publications. As a result, potentially valuable studies in other languages, particularly from regions with innovative digital sustainability solutions like China, Latin America, or Francophone Africa, may have been excluded. This introduces a degree of selection bias and may limit global generalizability.
- Publication Bias: As only peer-reviewed journal articles were considered, the review may have missed relevant insights from gray literature (e.g., government reports, NGO evaluations, technical white papers). This could skew findings toward academically validated interventions and away from grassroots or emerging innovations.
- Lack of Meta-Analysis: Due to heterogeneity in study designs, outcomes measured, and technologies evaluated, it was not feasible to conduct a quantitative meta-analysis. Instead, a narrative synthesis and thematic comparison were applied. While this provides rich qualitative insights, it limits statistical generalizability and effect size estimation.
- Variability in Outcome Reporting: Many studies lacked standardized metrics for either environmental or health impacts. For example, telehealth programs variously reported CO_2_ savings, travel distance, or PPE reduction, making cross-study aggregation difficult. Similarly, clinical outcomes ranged from broad access indicators to disease-specific clinical markers.
- Timeframe Constraints: This review focused on literature published between 2020 and 2025, aligning with the post-COVID acceleration of digital health. While this provides temporal relevance, it excludes pre-pandemic innovations and may overlook longer-term sustainability trends.
- Lack of Stakeholder Perspectives: This review did not directly assess user experience or provider perspectives on the usability or ecological awareness of these technologies. This is an important consideration for future research exploring behavioral drivers of adoption and long-term sustainability.

### 4.4. Research Gaps

While this review provides a comprehensive synthesis of both clinical and environmental outcomes of digital health interventions, several limitations must be acknowledged.

First, a quantitative meta-analysis was not conducted due to substantial heterogeneity in study designs, outcome measures, and reporting standards across the included studies. Specifically, many environmental outcomes such as reductions in energy use, carbon emissions, or waste were reported in narrative or anecdotal formats without standardized quantitative metrics or effect sizes. As a result, it was not possible to aggregate findings using statistical techniques or generate pooled estimates. This limits the ability to draw robust, generalizable conclusions about the magnitude of digital health’s environmental impact.

Environmental impact metrics used across studies varied widely. Some studies reported estimated CO_2_ savings per consultation, others discussed qualitative reductions in paper or PPE use, while very few employed standardized methodologies such as life-cycle assessment (LCA). The absence of consistent measurement frameworks presents a significant challenge in comparing outcomes across settings and technologies.

To enhance comparability and policy relevance, future research should aim to establish a unified framework for environmental evaluation of digital health interventions. Such a framework could include standardized indicators such as carbon footprint per patient encounter, digital device lifecycle emissions, energy use per virtual consultation, and reduction in healthcare waste volumes. These metrics should be integrated into digital health impact assessments alongside clinical performance indicators.

### 4.5. Environmental Externalities and the Need for Life-Cycle Perspective

While the environmental benefits of digital health technologies are increasingly documented, this review recognizes that digital interventions also introduce environmental costs that must not be overlooked. The production, deployment, and disposal of digital devices, including smartphones, wearable sensors, and cloud infrastructure, contribute to electronic waste (e-waste), which is often unmanaged in low- and middle-income countries. Additionally, energy-intensive data centers supporting telemedicine platforms, AI diagnostics, and electronic health records contribute to rising electricity demand, which may increase carbon emissions if not powered by renewable sources [26,59].

These negative externalities were acknowledged in several included studies but were often not explored in quantitative or systematic detail. Notably, only a minority of studies considered full environmental trade-offs across a technology’s life span. To address this gap, Life-Cycle Assessment (LCA) should be adopted more widely in future evaluations of digital health tools. LCA offers a holistic framework to assess upstream and downstream environmental impacts from raw material extraction and device manufacturing to usage energy and end-of-life disposal. This approach would support a more balanced and transparent evaluation of digital health’s sustainability performance [149].

Policymakers and technology developers should be cautious not to treat digital health as environmentally neutral or automatically sustainable. Without proper regulation, transparency standards, and renewable infrastructure, digital health systems could inadvertently exacerbate environmental degradation despite reducing emissions from physical care delivery.

### 4.6. Toward Actionable Policy Recommendations

Although this review underscores the importance of aligning digital health strategies with climate goals, it falls short of providing concrete, actionable policy guidance a gap that future work must address [150,151]. To advance from general advocacy to implementation, we propose the following practical policy recommendations based on our synthesis:

- Standardize environmental metrics in digital health evaluations (e.g., emissions per virtual consult, energy per gigabyte, device disposal rates).
- Mandate life-cycle sustainability reporting for digital health platforms, especially those deployed at scale through public health systems.
- Incentivize green design of digital devices through procurement policies that prioritize energy-efficient, recyclable, and modular hardware.
- Promote renewable-powered infrastructure for cloud services and data centers supporting digital health delivery.
- Support circular economy models, such as device refurbishing and recycling programs integrated into national e-waste policies.
- Invest in capacity building for LCA literacy among digital health implementers and public sector decision-makers.

As digital health becomes a cornerstone of global health delivery, the integration of sustainability indicators—such as carbon intensity per clinical encounter, resource consumption tracking, and device life-cycle emissions—should be standard in both public and private evaluation frameworks. These indicators will help ensure that digital health technologies do not simply shift emissions or costs elsewhere in the system but instead contribute to net environmental gains.

### 4.7. Future Research Directions

Despite recent advancements in digital health and increasing awareness of healthcare’s environmental footprint, this systematic review highlights several critical knowledge gaps that require urgent attention. Future research should prioritize multidimensional studies that address both clinical and ecological outcomes while ensuring inclusivity across income settings, technology types, and care models.

Table 6 outlines a strategic research roadmap for advancing sustainable digital health between 2025 and 2030. It prioritizes quantifying environmental impacts, optimizing health environment tradeoffs, and promoting equitable access in low-resource settings. The agenda aligns key technologies like telemedicine, AI, and wearables, with relevant SDGs to guide future interdisciplinary research and policy action.

#### 4.7.1. Need for Empirical Evidence on Environmental Impact

While digital health interventions such as telemedicine and wearables are assumed to reduce resource consumption and carbon emissions, few studies have employed rigorous environmental metrics. For instance, only a fraction of the reviewed studies conducted full life-cycle analyses (LCAs), energy audits, or carbon accounting. Future research should perform the following:

- Use standardized carbon footprint metrics (e.g., kg CO_2_-equivalent per patient interaction).
- Assess indirect environmental impacts (e.g., server energy, device manufacturing, disposal).
- Apply frameworks like ISO 14040 [152] for sustainability reporting.

#### 4.7.2. Cost–Benefit and Return-on-Investment Studies

To support decision-making by governments and health systems, future research should integrate environmental metrics into economic evaluations. This includes the following:

- Cost–benefit analyses comparing traditional vs. digital pathways (e.g., in chronic disease monitoring).
- Estimating long-term operational savings due to reduced waste, energy, and transportation.
- Quantifying intangible savings (e.g., fewer missed workdays, reduced burnout due to automation).

#### 4.7.3. Scaling in Low- and Middle-Income Countries (LMICs)

Most reviewed studies originated in high-income countries, yet LMICs bear the brunt of both healthcare access challenges and climate change impacts. Priority areas for LMIC research include the following:

- Evaluating the feasibility and sustainability of solar-powered mHealth or telemedicine in rural areas.
- Exploring mobile-first interventions that reduce reliance on hospital infrastructure.
- Building locally validated models for AI-based diagnosis, including computational efficiency in low-resource settings.

## 5. Conclusions

This systematic review highlights the dual potential of digital health technologies to enhance clinical outcomes while advancing environmental sustainability. By evaluating 68 studies from 2020 to 2025, we find that telemedicine, mHealth, wearables, and AI platforms contribute to reduced emissions, lower resource use, and improved healthcare access and quality. Telemedicine emerged as the most impactful intervention, especially post-COVID-19, offering both environmental and clinical advantages. Other tools like remote monitoring and AI-enabled diagnostics also demonstrated synergy between improved care and reduced environmental footprints. To achieve climate-aligned healthcare transformation, policy frameworks must move beyond digital adoption and include enforceable sustainability targets, life-cycle transparency, and environmental safeguards. However, key gaps persist, most notably the lack of standardized environmental metrics and the underrepresentation of LMICs. Future work should prioritize the development of standardized environmental metrics and life-cycle frameworks to enable consistent evaluation and comparison of digital health’s sustainability performance across contexts. In summary, digital health holds promise as a catalyst for climate-resilient and equitable healthcare systems. Strategic deployment and cross-sector collaboration will be essential to realizing this potential on a global scale.

## Figures and Tables

**Table 3:** It contrasts the current systematic review with prior reviews based on study designs, digital health technologies, and measured outcomes. It highlights which reviews addressed both health and environmental impacts, showcasing the broader scope of this review. Additionally, it documents the quality appraisal tools used, such as MMAT, CASP, and NOS, reflecting methodological diversity.

Paper	Study Designs Covered	Digital Tech Types	Health Outcomes	Environmental Outcomes	Quality Tool Used
This Review	Mixed	Telemed, mHealth, AI, Wearables, EHR	Yes	Yes	MMAT
[55]	Quant	Telemed, mHealth	Yes	No	NOS
[33]	Mixed	AI, EHR	Yes	No	CASP
This Review	Mixed	Telemed, mHealth, AI, Wearables, EHR	Yes	Yes	MMAT
[59]	Mixed	mHealth, Wearables	No	Yes	MMAT
[23]	Quant	Telemed	Yes	No	ROBINS-I
[21]	Quant	Wearables	No	Yes	CASP
[60]	Quant	AI	Yes	No	NOS
[61]	Quant	EHR, Telemed	Yes	Yes	AMSTAR
[14]	Quant	mHealth	No	Yes	CASP
[62]	Quant	Telemed, AI	Yes	No	NOS

**Table 4:** Summary of cross-domain synergies in digital health studies (2020–2025).

Technology	Health Outcome	Environmental Outcome	Setting	Year	Ref
Telemedicine	Improved chronic care and reduced wait times	Reduced transport emissions and PPE usage	Urban hospitals (USA)	2022	[123]
Wearables	Early detection of complications	Minimized lab test frequency	Geriatric clinics (Vietnam)	2023	[124]
AI Diagnostics	Improved diagnostic accuracy and reduced testing	Reduced re-tests and energy savings	Radiology units (Spain)	2020	[47]
mHealth Apps	Increased patient compliance	Avoided acute care burden	Community programs (Brazil)	2022	[125]
Blockchain Health Passports	Improved vaccine coverage	Reduced paper and in-person demand	Public health (Peru)	2023	[118]
Remote Monitoring	Reduced emergency admissions	Lower resource consumption	Cardiology home care (India)	2023	[113]
Chatbots	Improved therapy adherence	Reduced clinic visits	Adolescent mental health (Canada)	2023	[92]
Virtual Mental Health Platforms	Reduced depressive symptoms	Avoided in-person sessions	Schools and homes (Australia)	2022	[95]
Mobile Prenatal Care	Reduced maternal mortality	Avoided surgical interventions	Maternal clinics (Kenya)	2020	[126]

**Table 5:** Comparison of this review with key studies from 2020 to 2025.

Study	Scope	Key Findings	Limitations
This Review (2025)	Environmental and health outcomes of four digital health technologies (global scope)	Clear dual benefits across tech types; highlights synergy gaps and policy needs	Requires more LMIC-specific longitudinal data
[23]	Carbon footprint from telemedicine in US clinics	Confirmed 30% drop in CO_2_; limited patient outcome data	Narrow focus, lacks broad framework
[29]	Travel savings and access gains from rural telehealth	Improved rural access but lacked environmental metrics	Did not quantify ecological impact
[42]	Energy reduction from digital record systems	Moderate energy savings from digital transitions	Only administrative energy studied
[138]	Wearables for cardiac care in older adults	Clinical gains and reduced hospital days; low on sustainability metrics	No emission or supply chain metrics
[113]	Remote cardiac monitoring in India	Reduced ED admissions and logistics costs	No carbon quantified
[47]	AI in diagnostic triage and energy/resource efficiency	Reduced redundant testing and improved workflow	Focused only on radiology
[116]	mHealth app for smoking cessation outcomes	Behavior change + reduced resource use	Short-term only, no emissions data
[117]	Telemedicine with solar clinics in Bangladesh	Achieved maternal health impact + eco-savings	Small cohort study
[114]	AI triage impact in low-resource African hospitals	Avoided unnecessary care and emissions	Data from single-center pilot

**Table 6:** Future research agenda for sustainable digital health (2025–2030).

Research Area	Key Objective	Methodologies	Target Technologies	Relevance to SDGs
Environmental Impact Studies	Quantify emissions, energy, waste savings	LCA, carbon audits, energy modeling	Telemedicine, AI servers, wearables	SDG 13, SDG 12
Health–Environment Tradeoffs	Balance quality of care with ecological impact	Mixed methods, cluster trials, comparative modeling	mHealth, hospital EHRs, virtual wards	SDG 3, SDG 13
Cost–Benefit Evaluations	Economic + ecological ROI analysis	Cost-effectiveness + carbon valuation models	Remote monitoring, solar health hubs	SDG 8, SDG 9
Equity in LMICs	Sustainable tech access in rural/low-resource areas	Community trials, stakeholder interviews	Mobile apps, solar-powered diagnostics	SDG 10, SDG 3
Interoperability + Systems Integration	Streamline platforms to avoid resource duplication	Systems engineering, health informatics mapping	EHRs, AI-enabled triage, IoT platforms	SDG 9, SDG 17
Behavioral Drivers	Understand adoption and eco-awareness	Surveys, digital ethnography, co-design	All digital tools (cross-cutting)	SDG 3, SDG 11

## Data Availability

No new data were used in this study. The review has been registered on Open Science Framework on 28 July 2025 with the following DOI: https://doi.org/10.17605/OSF.IO/M7AJX.
